# Biomedical optical imaging of acne pathology: from single-modality methods to multimodal integration

**DOI:** 10.3389/fmed.2026.1710354

**Published:** 2026-04-28

**Authors:** Xing Ren, Jing Yang, Yanan Sun, Qianghua Quan, Peilin Zhang, Yunong Wang, Yi Wang

**Affiliations:** 1 Yunnan Baiyao Group Co., Ltd., Kunming, China; 2 Beijing Changping Hospital of Integrated Chinese and Western Medicine, Beijing, China; 3 Experimental Research Center, China Academy of Chinese Medical Sciences, Beijing, China; 4 Beijing Cowisdom Biotechnology Co., Ltd., Beijing, China; 5 Beijing University of Chinese Medicine Third Affiliated Hospital, Beijing, China

**Keywords:** acne vulgaris, bio-optical signals, multimodal, optical imaging, pathology

## Abstract

Acne vulgaris is a prevalent inflammatory skin disorder, and the objective, timely, and accurate assessment of its severity is critical for guiding standardized treatment. Conventional evaluation methods are often subjective and time-consuming, limiting clinical efficiency. Optical imaging technologies, characterized by non-invasiveness and high resolution, enable the rapid visualization of key pathological features at the microstructural level, including follicular hyperkeratinization, sebaceous hyperactivity, bacterial colonization, and inflammation. Single-modality imaging techniques, such as OCT, RCM, dermoscopy, LSCI, UV fluorescence imaging, and HSI, can delineate various acne-related changes but face limitations in complex clinical settings. In contrast, multimodal imaging integrates complementary techniques to offer a more comprehensive pathological profile, demonstrating significant potential in clinical applications. Future advancements should prioritize enhanced resolution, sensitivity, and integration with artificial intelligence to improve the precision and reliability of acne severity assessment. This review summarizes the photobiological properties of key acne-related biomarkers and their potential to be harnessed through optical imaging for improved diagnosis and management.

## Introduction

Acne vulgaris is a chronic inflammatory disorder of the pilosebaceous units (1, 2). It affects approximately 9% of the global population and up to 85% of adolescents, thereby exerting considerable psychological, social, and economic burdens on affected individuals (3, 4). Accurate and timely assessment of acne severity is critical for dermatologists to formulate standardized and effective treatment strategies. Commonly employed scoring frameworks include the Global Acne Grading System (GAGS), which assigns composite severity scores based on lesion type and density across six anatomical regions (5); the Investigator’s Global Assessment (IGA), a five-point ordinal scale widely adopted in regulatory clinical trials for its inter-rater reliability (6); and direct lesion counting, which enumerates individual comedones, papules, pustules, and nodules to yield quantitative burden data (7). Despite their structured design, all three frameworks depend substantially on visual inspection and subjective interpretation. In particular, manual lesion counting is labor-intensive, time-consuming, and susceptible to interobserver variability, thus limiting its clinical utility (8).

In recent years, skin imaging technologies have emerged as a key research focus for the assessment of acne severity. Among these approaches, the integration of image processing techniques with machine learning has facilitated more efficient and objective diagnosis of facial acne. Deep learning algorithms have been extensively employed in image classification and lesion detection tasks. Shen et al. (9) used the VGG16 model to classify six types of acne lesions—blackheads, whiteheads, papules, pustules, nodules, and cysts—as well as normal skin, enabling a more detailed analysis of overall facial acne. Yang et al. (10) adopted Inception-v3 as the backbone of their deep learning model and developed a severity assessment method that integrates clinical features with treatment strategies based on Chinese guidelines for acne treatment. Compared to VGGNet and ResNet, Inception-v3 performs better in detecting subtle differences while offering lower computational cost. Wang et al. (11) designed a lightweight deep learning model, Acne-RegNet, based on the GAGS and Hayashi criteria, and deployed it on mobile devices to enable real-time severity assessment and treatment recommendations. Zhang and Ma (12) proposed an acne detection approach based on ensemble neural networks, capable of simultaneously assessing acne severity, lesion count, and spatial distribution. Deep learning-based methods have demonstrated significant advancements in the detection and grading of acne. However, challenges such as limited dataset size and diversity, along with inadequate model robustness under variable conditions, have reduced classification accuracy and hindered the clinical applicability of these methods in complex scenarios. More recently, multimodal large language models (LLMs) have also been applied to clinical image-based acne assessment: GPT-4o demonstrated high accuracy in primary acne diagnosis, but substantially lower performance in lesion subtype classification, with comparable patterns of diagnostic promise observed when LLMs were applied to the related pilosebaceous inflammatory condition hidradenitis suppurativa (13, 14). Critically, these approaches are largely confined to macroscopic surface appearances captured by standard photography and do not interrogate the sub-surface pathophysiological signals that underlie acne progression. In contrast, bio-optical imaging modalities aim to capture mechanistic and structural signals that are directly coupled to the underlying pathophysiology of acne.

Investigating deeper skin structures and features related to the pathophysiology of acne has emerged as a prominent research frontier. The pathogenesis of acne is multifactorial, encompassing excessive sebum production, follicular hyperkeratinization, *Propionibacterium acnes* (*P. acnes*) proliferation, and inflammation (15). The progression of acne from microcomedones to inflammatory lesions represents a complex, multidimensional, and dynamic biological process. Throughout this process, acne-associated pathological factors induce structural and compositional alterations in the skin (16, 17). Optical imaging technologies, owing to their non-invasive nature, minimal tissue disruption, and high spatial resolution, have been widely adopted for the assessment of skin architecture and pathological features. Cutaneous erythema intensity is quantifiable via spectroscopic or polarized-light methods (18); follicular plugging morphology and comedone depth are resolvable with high-resolution optical coherence tomography (OCT) (19); microvascular perfusion dynamics are mappable through laser speckle contrast imaging (LSCI) or Doppler-based techniques (20); and subclinical peri-follicular inflammatory infiltrates not visible to the naked eye are detectable by reflectance confocal microscopy (RCM) (21). These technologies are capable of capturing pathological signals directly associated with acne pathogenesis and offer objective, reproducible insights into intradermal morphological alterations (22, 23). Nonetheless, global severity integration across body regions continues to require clinical judgment and cannot be fully replaced by imaging-derived metrics alone.

This review aims to examine key bio-optical signals implicated in the pathophysiology of acne, along with the corresponding imaging modalities. In addition, it outlines the current clinical applications of these imaging approaches in acne management and highlights the need to transition from single-parameter analysis to multidimensional integration in optical imaging-based assessment of acne severity.

## Bio-optical signals in acne pathology

**Figure 1:**
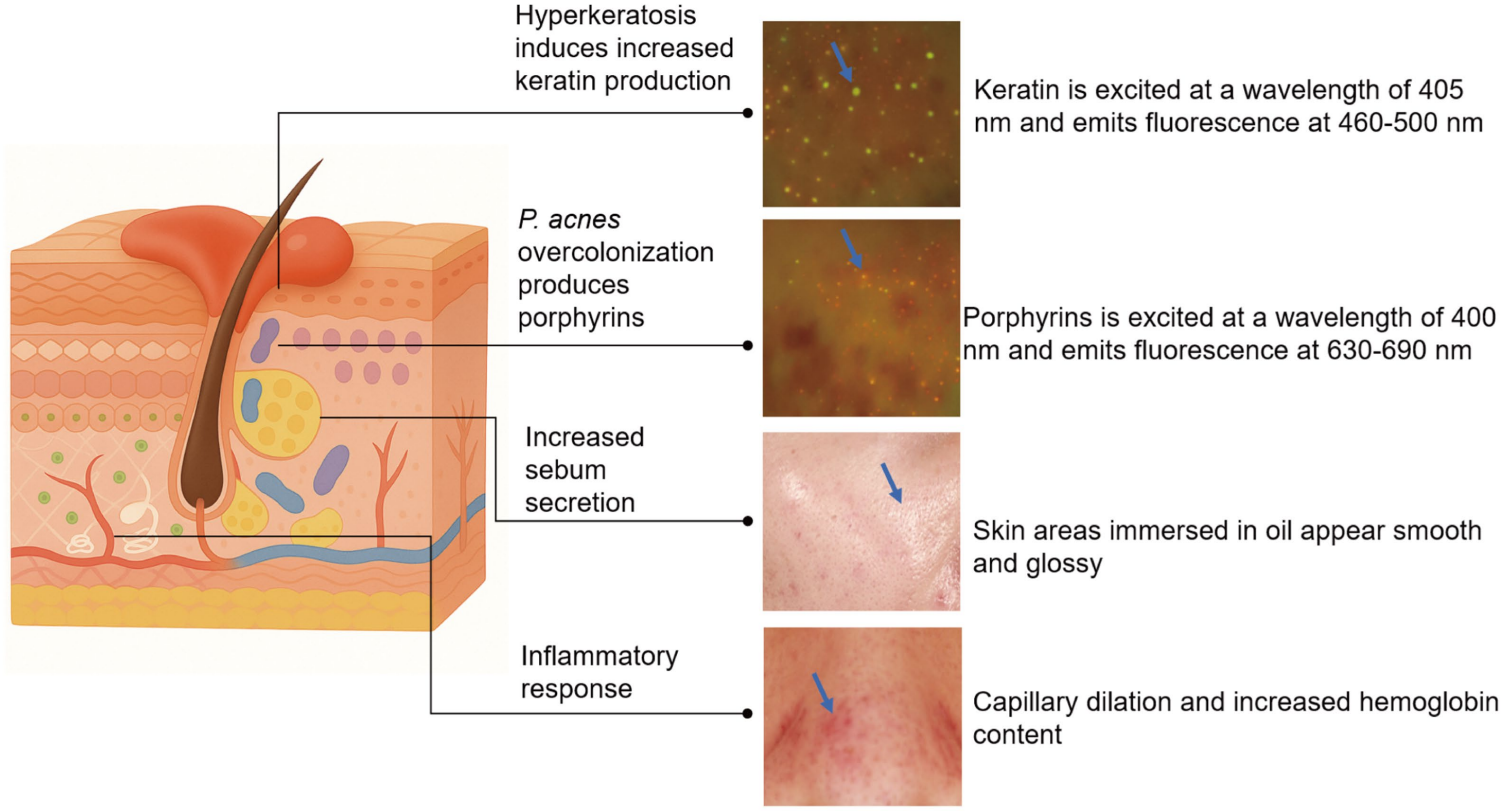
Schematic of bio-optical signals in acne pathology.

The pathological progression of acne represents a dynamic, multifactorial process that disrupts the structural integrity, morphology, and homeostatic balance of skin tissue (24). Acne is generally believed to originate with the formation of microcomedones, which subsequently develop into comedones or inflammatory lesions. The formation of microcomedones results from multiple pathogenic factors, including excessive sebum secretion and/or altered lipid composition, abnormal keratinization of the follicular duct, and bacterial colonization (25, 26). Microcomedones consist of dilated follicles filled with microscopic keratin flakes, bacteria, and sebum (3). The biofilm formed by *P. acnes* acts as a biological adhesive, promoting the aggregation of desquamated keratinocytes and sebum, ultimately leading to comedone formation (27, 28). Colonization of hair follicles by *P. acnes*, along with biofilm formation, initiates inflammatory cascades and immune cell recruitment, ultimately resulting in inflammatory acne lesions including papules, pustules, cysts, and nodules (29–32). The physiological, morphological, and biochemical characteristics of the skin give rise to distinct optical responses involving light absorption, scattering, and emission. These optical responses can be used for quantification, analysis, and visualization, offering a direct and intuitive means to observe the progression of acne lesions (Figure 1) (33). To facilitate clinical translation, Table 1 summarizes the optical properties and most suitable imaging modalities for all four biomarkers. The following section discusses the optical signatures associated with acne pathological changes.

**Table 1:** Summary of key acne-related bio-optical biomarkers.

Biomarker	Optical signature	Excitation/absorption peak(s)	Emission peak(s)	Key wavelength range	Most suitable modality
Keratin *Follicular keratin plug; stratum corneum accumulation*	↑ Autofluorescence; ↑ Light scattering (local reflectance due to denser corneocyte packing)	395–405 nm	460–500 nm	UVA to blue visible (365–500 nm)	UV fluorescence imaging RCM OCT Multiphoton (TPEF)
Sebum *Surface lipids and sebaceous gland content; lipid-rich follicular plug*	↓ Surface reflectance in visible; ↑ Specular reflectance in NIR; low-scattering gland voids in OCT	Not applicable	Not applicable	Visible (400–600 nm, negative correlation); NIR (700–2,500 nm, positive correlation)	Polarized reflectance imaging OCT
Coproporphyrin III/Porphyrins *P. acnes-derived endogenous porphyrins; coproporphyrin III predominant subtype*	↑ Fluorescence; Orange-red fluorescent spots co-localize with comedone/inflammatory lesion distribution	320–400 nm	Coproporphyrin III: 570–630 nm	UVA excitation (~365 nm); Emission: orange-red visible (570–690 nm)	UV fluorescence imaging (Wood’s lamp); VISIA-CR.
Oxyhemoglobin (HbO₂) and Deoxyhemoglobin (Hb) *Marker of vasodilation and perfusion in inflammatory lesions*	Strong wavelength-dependent absorption; ↑ Erythema with total Hb in inflammatory lesions	HbO₂ Abs: 542 nm and 577 nm; Hb Abs: ~555 nm (lower); Hb > HbO₂ beyond 600 nm	Not applicable	Green–red visible (500–700 nm); Isosbestic point ~530 nm and ~800 nm	LSCI Hyperspectral Imaging (HSI); Dynamic OCT (D-OCT); Diffuse reflectance spectroscopy

Ex = excitation peak; Em = emission peak; Abs = absorption peak; NIR = near-infrared.

## Hyperkeratinization increases keratin autofluorescence

In healthy skin, the stratum corneum exhibits a relatively uniform scattering coefficient, with light scattering primarily attributed to corneocytes and their organized structural arrangement. However, with the onset of follicular hyperkeratinization, keratinocyte proliferation becomes abnormal, resulting in the thickening of the stratum corneum and a denser cellular arrangement. Electron microscopy reveals an increased number and size of keratohyalin granules, along with a significant rise in tonofilaments and desmosomes. The accumulation of these dense intracellular structures enhances light scattering, thereby increasing local reflectance (16, 34). During hyperkeratinization, keratinocytes begin to accumulate within the epidermis, accompanied by an increase in keratin production. Ki67, keratin 6 (K6), and keratin 16 (K16) are phenotypic markers of keratinocyte hyperproliferation and abnormal differentiation (35). Notably, even some “normal” follicles in acne-prone skin may exhibit overexpression of Ki67 and K16 (36). Keratin is also one of the skin’s endogenous fluorophores, with peak excitation at 405 nm and fluorescence emission between 460 and 500 nm. Under 395 nm excitation, the accumulated keratin autofluorescence creates strong contrast with the surrounding skin (37). The autofluorescent properties of keratin are primarily attributed to the presence of aromatic amino acid residues, particularly tyrosine and tryptophan (38). These aromatic residues emit fluorescence when excited by ultraviolet (UV) light. Within the molecular structure of keratin, these residues are often located in specific microenvironments that enhance their sensitivity to UV excitation and fluorescence emission. For example, open comedones emit a yellow-white fluorescence under UVA exposure due to keratin plugs, whereas closed comedones typically do not fluoresce (39).

## Excessive sebum alters skin optical reflectance

The role of excessive sebum production in the pathophysiology of acne has been well established. Early studies revealed that acne patients produce significantly more sebum than individuals without acne (40–43). Excessive sebum disrupts the follicular barrier, promoting comedone formation, overgrowth of *P. acnes*, and inflammation, ultimately contributing to acne development (44, 45). Sebum levels are correlated with the skin’s directional reflectance. In the visible light range (400–540 nm and 480–600 nm), sebum content is negatively correlated with directional reflectance, indicating that higher sebum levels lead to greater absorption or transmission of radiation into the skin (46). This phenomenon is attributed to the composition of surface skin lipids—including fatty acids, triglycerides, wax esters, sterol esters, squalene, and paraffin—which have refractive indices ranging from 1.44 to 1.5, higher than the natural refractive index of skin (~1.33). As a result, the presence of surface lipids alters the skin’s optical properties by increasing light transmission and diffuse reflection while reducing surface reflectance. When sebum fills pores and wrinkles, it smooths the skin surface, narrowing the angle of surface reflectance. Skin areas partially immersed in oil appear blotchy, irregular, and shiny, whereas fully immersed areas appear smooth and glossy. Based on this principle, differential measurements of surface and diffuse reflectance within the visible spectrum enable non-contact, objective quantification of skin greasiness, offering an effective tool for acne diagnosis and monitoring (47). In contrast, in the near-infrared (NIR) regions—specifically 590–720 nm, 700–1,100 nm, 1,000–1,700 nm, and 1,700–2,500 nm—sebum content shows a positive correlation with skin specular reflectance. This indicates that increased sebum leads to higher reflectance in these bands, suggesting a potential protective effect of sebum against NIR radiation (46). Sebum production is primarily determined by sebaceous gland function. Sebaceous glands are typically located in the dermis and appear as oval or spherical low-scattering regions or voids. This low-scattering characteristic is due to the weak light-scattering capacity of the lipid-rich content within the glands. This optical property facilitates the clear distinction of sebaceous glands from surrounding skin structures in optical coherence tomography (OCT) images (48).

## Porphyrins produced by *P. acnes* can emit fluorescence under ultraviolet light

*P. acnes* is a highly abundant bacterial species in the skin microbiome, predominantly residing in sebaceous regions such as the face and back, which are typically anaerobic and lipid-rich (49). Studies have shown that porphyrins produced by *P. acnes* are closely associated with inflammatory skin conditions, particularly acne, with significantly higher porphyrin levels observed in the skin of acne patients than healthy individuals (50). Porphyrins contribute to perifollicular inflammation through their cytotoxic effects and by stimulating IL-8 expression in keratinocytes (2, 51). Additionally, porphyrins interact with keratinocyte membranes, inducing potassium efflux, which activates the NLRP3 inflammasome and triggers IL-1β release (52). Therefore, porphyrins play a critical role in the pathogenesis of acne, potentially initiating or exacerbating inflammatory responses through multiple mechanisms. Porphyrins are endogenous fluorophores with distinct fluorescence properties, exhibiting peak excitation around 400 nm and emission in the 630–690 nm range (53). The structural features of porphyrins—particularly their conjugated double-bond systems and cyclic arrangement of four pyrrole rings linked by methine bridges—facilitate electronic transitions and fluorescence emission (54, 55). Studies have found that porphyrin production by *P. acnes* is significantly elevated in acne-affected skin compared with that in normal skin, with coproporphyrin III being the predominant subtype (56). Coproporphyrin III exhibits strong fluorescence under ultraviolet A (UVA) irradiation, with emission wavelengths ranging from 570 to 630 nm (57–59). The spatial distribution of its fluorescent spots shows a strong correlation with comedone lesion counts (60). This suggests that coproporphyrin III fluorescence may serve as a sensitive and specific biomarker for *P. acnes*, particularly valuable for evaluating treatment efficacy and detecting early signs of acne progression. Nevertheless, it is important to interpret UV-induced porphyrin fluorescence as a useful but inherently imperfect surrogate. Several lines of evidence support this qualification. Its clinical value, therefore, lies in providing complementary, semi-quantitative information within a multimodal assessment framework, rather than serving as a standalone, lesion-specific diagnostic index.

## Inflammation induces hemoglobin accumulation and vasodilation

Inflammation during acne lesion formation affects exocytosis and vascular proliferation, leading to local capillary dilation and increased perfusion. Clinically, this manifests as erythema, which results from a significant increase in hemoglobin concentration (61, 62). Hemoglobin is a major chromophore responsible for skin color and exists in two forms within the dermal microvasculature: oxyhemoglobin and deoxyhemoglobin (63). Oxyhemoglobin exhibits peak absorption at 542 nm and 577 nm, with a sharp decline in its extinction coefficient beyond 600 nm. Deoxyhemoglobin has lower absorption at 555 nm, but its absorption exceeds that of oxyhemoglobin beyond 600 nm (64). Therefore, the concentrations of oxyhemoglobin and deoxyhemoglobin can be calculated by quantifying the spectral reflectance of skin samples (65). This suggests that the severity of inflammation in acne lesions can be evaluated by quantitatively analyzing hemoglobin. Additionally, during acne-associated inflammation, there is a significant increase in the NADPH autofluorescence signal in epidermal granular layer cells (66). Nicotinamide Adenine Dinucleotide Phosphate (NADPH) exhibits intrinsic autofluorescence, primarily in its reduced form, whereas its oxidized form (NADP^+^) lacks this property. This fluorescence property makes NADPH an important biomarker for studying intracellular metabolic activity and redox state (67). Therefore, the intensity of NADPH autofluorescence in granular layer cells can serve as a biomarker for detecting the inflammatory state in acne.

## Application of optical imaging in acne pathology characterization

### Single-modality optical imaging technology

In the pathological characterization of acne, single-modality optical imaging techniques have been widely utilized due to their unique advantages. These techniques include optical coherence tomography (OCT), reflectance confocal microscopy (RCM), dermoscopy, laser speckle contrast imaging (LSCI), UV-induced fluorescence imaging, and hyperspectral imaging (HSI). Each modality is based on a distinct imaging principle and is capable of capturing specific imaging features associated with acne lesions, thereby providing valuable information for diagnosis, severity grading, and treatment monitoring. However, these techniques also have their limitations, which may hinder their broader application in clinical practice. Table 2 summarizes the application of various single-modality imaging techniques in assessing pathological changes in acne.

**Table 2:** Characterizing acne pathology with single-modality optical imaging.

Optical imaging technology	Imaging principle	Imaging characteristics	Acne-related pathological changes
OCT/D-OCT (23, 78, 87–91)	Cross-sectional images are generated by collecting reflected and backscattered infrared light.	Hypoechoic structures with an inverted V-shape or rectangular boundaries are often observed, with hyperechoic regions frequently present between the two hypoechoic margins. The dermal vascular network surrounding the lesion is significantly enhanced.	Thickening of the stratum corneum and follicular hyperkeratinization lead to the formation of keratin plugs Infiltration of inflammatory cells
RCM (16, 17, 78, 92)	The black-and-white images are generated based on the varying refractive properties of different skin structures.	The borders of the follicular infundibulum are expanded, appearing as irregular, hyper-reflective structures in dark gray or white. Poorly defined, round-like areas contain bright granules, small reflective inflammatory cells, and densely packed, bright amorphous materials with organized structures.	Thickening of the stratum corneum Keratin accumulation Infiltration of inflammatory cells
Dermoscopy (72, 89, 93, 94)	High-magnification microscope objectives are used to enlarge the surface and superficial microstructures of the skin.	The pilosebaceous units are filled with white-yellow circular structures or brown plugs. Inflammatory acne lesions appear as round structures with a white center, a thin brown border, and surrounding erythema.	Follicular hyperkeratinization and capillary dilation lead to erythema formation.
LSCI (23, 61, 74, 95–97)	When laser radiation is directed onto the skin surface, the laser beam undergoes random scattering within the tissue, resulting in the formation of a speckle pattern.	Blood perfusion in acne lesions is significantly increased.	Increased chemotaxis of inflammatory cells
Ultraviolet fluorescence imaging (58, 60, 84, 98, 99)	By emitting long-wave ultraviolet radiation (UVR), certain skin components absorb the UV light and re-emit it as visible fluorescence.	The skin exhibits orange-red or yellow-white fluorescence.	*P. acnes* colonization leads to porphyrin secretion Follicular hyperkeratinization results in dense keratin aggregation
HSI (61, 76, 77, 100)	The incident light is decomposed into dozens or even hundreds of spectral bands, providing rich spectral information for each pixel.	The reflectance of acne lesions is significantly reduced in the 400–600 nm wavelength range.	Hemoglobin concentration increases in inflamed acne lesions *P. acnes* produce elevated levels of porphyrins

OCT is a well-established, non-invasive diagnostic tool with a lateral resolution of 3–15 μm and a penetration depth of 1–2 mm. Dynamic OCT (D-OCT) visualizes blood flow changes in the epidermis, dermis, and associated microvasculature, enabling morphological and functional characterization of skin lesions (68–70). In acne pathology, OCT visualizes both thickening of the stratum corneum and the keratin plugs formed by follicular hyperkeratinization. Furthermore, D-OCT can visualize microvascular blood flow changes in the skin in real-time, providing crucial information for assessing inflammatory acne. Clinically, OCT—particularly D-OCT—is best deployed for depth-resolved structural characterization, including assessment of sebaceous gland morphology, stratum corneum thickening, and real-time microvascular perfusion changes. It is therefore most valuable in the monitoring of moderate-to-severe inflammatory acne, evaluation of nodular or cystic lesion depth, and longitudinal assessment of scarring risk before and after treatment. However, OCT is insensitive to epidermal metabolic activity and bacterial viability, resulting in insufficient sensitivity for detecting early-stage inflammation.

RCM uses an 830-nm diode laser as a point light source to capture en face images of the skin. It provides histology-level resolution and penetrates to a depth of 200–300 μm, covering the entire epidermis and the dermal papillary layer (71). RCM can identify abnormalities in the number of corneocyte layers and allows quantitative assessment of the follicular infundibulum, including its diameter, boundaries, contents, and the density of inflammatory cells. In acne lesions, RCM reveals characteristic features, including dilation of the follicular infundibulum, keratin accumulation, and infiltration of inflammatory cells. RCM is best suited to scenarios requiring cellular-level microstructural detail in superficial skin layers. However, RCM is limited by its imaging depth, which does not extend beyond the epidermis and papillary dermis. Image interpretation is somewhat subjective, and both image acquisition and analysis are time-consuming, making it unsuitable for rapid screening of large numbers of patients in clinical practice.

Dermoscopy is a non-invasive diagnostic technique that enables visualization of subtle structures in both the superficial and deeper layers of the skin. It employs magnification ranging from ×10 to ×100, combined with a light source, to visualize subsurface structures such as melanin and blood vessels (72). In acne lesions, dermoscopy can reveal follicular keratin plugs, abnormal vascular patterns (such as arborizing capillaries), and pigmentation distribution. However, dermoscopy lacks quantitative analysis capability, and diagnosis largely depends on clinical expertise and subjective interpretation. Moreover, dermoscopy cannot provide information on subcutaneous structures, limiting its utility in evaluating deep inflammation or scarring. Despite these limitations, dermoscopy remains the most practical first-line imaging tool for rapid bedside triage in acne.

LSCI is a real-time imaging technique used to visualize microcirculation within tissues. When laser radiation is directed at the skin surface, the beam undergoes random scattering within the tissue, generating a speckle pattern. This speckle pattern results from the scattering of laser light by microscopic particles in the skin, such as red blood cells (73, 74). In acne lesions, LSCI can monitor changes in blood perfusion in real time, allowing assessment of local vasodilation and response to anti-inflammatory treatment. LSCI is best employed in scenarios that require wide-field, real-time monitoring of cutaneous perfusion dynamics. However, LSCI has relatively low spatial resolution and is susceptible to motion artifacts from skin surface movement. Additionally, LSCI provides only relative blood flow velocity, making it unsuitable for precise quantification of absolute blood flow. Its ability to resolve microvascular structures is also limited.

Ultraviolet fluorescence imaging is based on the emission of long-wave UV light, primarily within the 320–400 nm range, with a peak wavelength at 365 nm. In acne lesions, ultraviolet fluorescence imaging detects the characteristic fluorescence of porphyrins under UV light, typically appearing as orange-red signals, to localize bacterial colonization and areas of inflammatory activity within hair follicles (75). UV fluorescence imaging is most valuable as a wide-field screening tool for mapping the distribution of follicular porphyrin accumulation across the face. However, the correlation between fluorescence intensity and disease severity remains controversial. In addition, the potential phototoxicity of UV exposure limits its use for repeated or long-term imaging.

HSI is an advanced imaging technique that captures continuous spectral information across the visible to near-infrared range. It decomposes incoming light into multiple spectral bands, providing rich spectral data for each pixel, thereby enabling detailed analysis of the target (76, 77). In acne lesions, HSI enables label-free differentiation between inflammatory and non-inflammatory subtypes through multi-wavelength spectral analysis. HSI is best suited to research where multi-parameter, label-free tissue characterization is the priority. However, HSI generates high-dimensional data that requires complex dimensionality reduction and modeling algorithms, resulting in poor real-time performance. In addition, HSI systems are expensive and highly susceptible to ambient light interference, limiting their widespread clinical adoption.

### Multimodal optical imaging technology

With the continuous advancement of optical imaging technologies, multimodal optical imaging has shown great potential in the diagnosis, grading, and treatment monitoring of acne. By integrating multiple imaging modalities, it provides more comprehensive and in-depth pathological information, thereby offering stronger support for the diagnosis and management of acne. Fuchs et al. (78) combined RCM and OCT to visualize the follicular infundibulum morphology, acne lesions, and local blood flow. Manfredini et al. (79) used RCM and dynamic OCT (D-OCT) to characterize subclinical and microscopic features of acne-prone skin and to evaluate microstructural changes induced by topical treatments. The combined use of RCM and OCT helps overcome the resolution–penetration trade-off, allowing simultaneous capture of both macrostructures and microscopic details. This enables the acquisition of both structural and functional skin information, providing a more comprehensive assessment of acne lesions. However, the combined use of OCT and RCM requires simultaneous access to both systems, which increases equipment costs and operational complexity.

The VISIA-CR system integrates multiple imaging techniques, including standard white-light imaging, ultraviolet fluorescence, parallel-polarized, and cross-polarized light imaging, to quantitatively analyze various skin features. This multimodal imaging approach provides more comprehensive information on acne lesions, including the detection of both inflammatory and non-inflammatory lesions, and the quantification of erythema and hyperpigmentation (80). The VISIA-CR system represents a considerably more operationally accessible multimodal platform: a standardized full-face acquisition across all illumination modes (white light, UV fluorescence, parallel- and cross-polarized) is completed in under 5 min with minimal operator training; the system occupies a compact footprint of approximately 0.5 m^2^; and its automated image capture and analysis pipeline substantially reduces the burden of operator expertise. These characteristics make VISIA-CR compatible with integration into routine dermatology clinic workflows, and account in large part for its status as the only multimodal optical imaging system currently deployed in commercial clinical practice for acne assessment.

Table 3 summarizes how optical signals obtained under different illumination modes are used to grade acne severity and evaluate the efficacy of acne treatments. These bio-optical signals have been clinically validated for characterizing the pathological changes in acne. Patwardhan et al. (81) used the VISIA-CR multimodal imaging system to detect and classify acne lesions, offering an accurate, reproducible, and clinically relevant method for acne assessment. The system significantly reduces the laborious process of manual lesion counting and delivers lesion counts and localization comparable to those of expert dermatologists. The VISIA-CR system also provides quantitative measurements of lesion-specific inflammation, residual erythema, and post-acne hyperpigmentation, facilitating a more comprehensive evaluation of acne severity. However, limitations remain in the biological interpretation of the optical signals, and the quantification of pathological features is often imprecise. In addition, there is currently no robust quantitative algorithm for processing and analyzing image data to integrate optical signals obtained under different illumination modes for acne severity assessment.

**Table 3:** Application of VISIA-CR in the diagnosis and management of acne.

Illumination mode	Bio-optical signal	Pathological characterization	Clinical application
Standard white-light imaging	Original photograph	Acne lesions	The efficacy of the cosmetic product (IP/SG) on mild-to-moderate facial acne was evaluated by counting different types of lesions. (101)
Ultraviolet fluorescence imaging	Skin fluorescence	Porphyrin levels	The amount of fluorescence reflects acne severity and can be used to evaluate the effectiveness of drugs in inhibiting *P. acnes*. (39, 102)
Parallel-polarized imaging	Skin gloss	Sebum content	A quantitative method for measuring skin surface oiliness was developed by performing differential analysis between surface and subsurface reflections using parallel and cross-polarized images. (47)
Cross-polarized imaging	Skin erythema	Hemoglobin concentration	The combination of clindamycin 1% and tretinoin 0.025% was evaluated for its superior efficacy and tolerability compared to either monotherapy with benzoyl peroxide 5% or a fixed-dose combination of clindamycin 1% and benzoyl peroxide 5%. (103)

Despite the promise of multimodal integration, a fundamental and frequently underappreciated barrier to its clinical translation lies in the challenge of co-registration and cross-modality standardization. Each imaging modality operates under distinct physical constraints—including differences in field-of-view, depth sampling range, illumination angle and geometry, and spatial resolution—that make the spatial alignment of complementary datasets technically non-trivial. Motion artifacts introduced between sequential acquisitions, particularly for contact-based modalities such as RCM, can further disrupt the anatomical correspondence necessary for meaningful signal fusion. Time intervals between acquisitions of different modalities may additionally introduce biological variability, particularly for dynamic signals such as microvascular perfusion measured by LSCI or D-OCT. Addressing these challenges will require the field to adopt a set of practical co-registration strategies.

At the hardware level, fiducial marker systems and standardized facial mapping grids—analogous to those used in longitudinal dermatology photography—can provide stable anatomical reference frames across imaging sessions and modalities. Dedicated dual-modality probes that physically co-locate two imaging systems (such as combined RCM-OCT probes currently in development) eliminate inter-acquisition motion for paired modalities. At the software level, deformable image registration algorithms trained on skin tissue deformation patterns and illumination-normalization pipelines that correct for geometry-dependent intensity variation are essential prerequisites for reliable feature fusion. Beyond technical solutions, the field currently lacks consensus on minimum reporting standards, which severely limits cross-center comparability of multimodal acne imaging studies. We therefore propose that future studies should, at minimum, report: (i) the spatial co-registration method employed and its estimated alignment error; (ii) the time interval between acquisition of each modality; (iii) the illumination geometry and device settings for each channel; (iv) the facial landmark or mapping system used to ensure anatomical reproducibility across sessions; and (v) the severity scale against which imaging-derived parameters are correlated. Establishing such reporting norms is a prerequisite for meta-analytic synthesis of multimodal imaging data and for the regulatory-grade evidence that clinical deployment ultimately requires.

## Discussion

With the rapid advancement of optical imaging technologies, multimodal imaging systems have demonstrated great potential in assessing acne severity and evaluating treatment efficacy. Traditional single-modality imaging techniques, such as OCT, RCM, and dermoscopy, provide valuable structural, functional, and molecular insights into acne pathology. However, their inherent limitations—such as limited information dimensionality and the trade-off between imaging depth and resolution—restrict their use in complex clinical settings. By integrating multiple imaging modalities, multimodal systems can simultaneously capture various types of image data—such as red-area, fluorescence, and brown-area images—providing multidimensional information about acne lesions. This enables a more comprehensive evaluation of acne severity.

However, the biological significance of the multidimensional optical signals obtained from multimodal imaging systems remains to be fully elucidated. Although UVA-induced facial red fluorescence (UVRF) has long been attributed to coproporphyrin III produced by *P. acnes* (82, 83), the reliability of this interpretation remains controversial (58, 84, 85). In fact, additional information embedded within fluorescence images has yet to be explored. Beyond orange-red fluorescent spots, keratin-associated fluorophores can emit white fluorescence under 370 nm excitation (86). Therefore, it is essential to investigate additional optical signals in multimodal imaging and to clarify their specific biological implications.

In future, the application of multimodal imaging in acne severity assessment should focus on improving imaging resolution and sensitivity, further optimizing imaging techniques to enhance the detection of early inflammation and subtle lesions. Integrating artificial intelligence and machine learning to develop more intelligent image analysis algorithms, thereby improving the accuracy of lesion classification and quantitative analysis. Integrating diverse imaging modalities enables more comprehensive acquisition of dermatopathological information, while streamlining imaging workflows to reduce equipment costs and operational complexity. At the hardware level, co-registered acquisition systems that spatially align complementary modalities in a single imaging session—for example, combining RCM for cellular-resolution mapping of follicular keratin plugs and inflammatory cell infiltration with D-OCT or LSCI for concurrent perfusion imaging—would enable disambiguation of active inflammation from post-inflammatory erythema that neither modality can resolve alone. At the software level, feature fusion algorithms that extract and jointly model complementary optical signals—such as integrating UV fluorescence-derived porphyrin distribution maps with cross-polarized reflectance images to separate genuine bacterial fluorescence from surface sebum shine—would substantially reduce signal ambiguity and improve the specificity of automated lesion classification. At the clinical integration level, multimodal imaging workflows must be structured to support concrete diagnostic decisions: for instance, a triage pathway in which wide-field UV fluorescence screening identifies follicular porphyrin burden, followed by targeted RCM for subclinical inflammation confirmation, and LSCI for treatment response monitoring, would translate multimodal data into a sequential, actionable clinical workflow rather than a parallel data collection exercise. Advancing integration across all three levels, while streamlining workflows to reduce equipment costs and operational complexity, represents the central engineering and translational challenge for the next generation of optical imaging-based acne assessment systems.

## Conclusion

In summary, optical imaging technologies have played a critical role in characterizing the pathological changes in acne, particularly multimodal imaging, which has enabled a shift from single-parameter analysis to multidimensional data integration and holds promise as a powerful tool for future acne diagnosis, severity assessment, and treatment evaluation.
